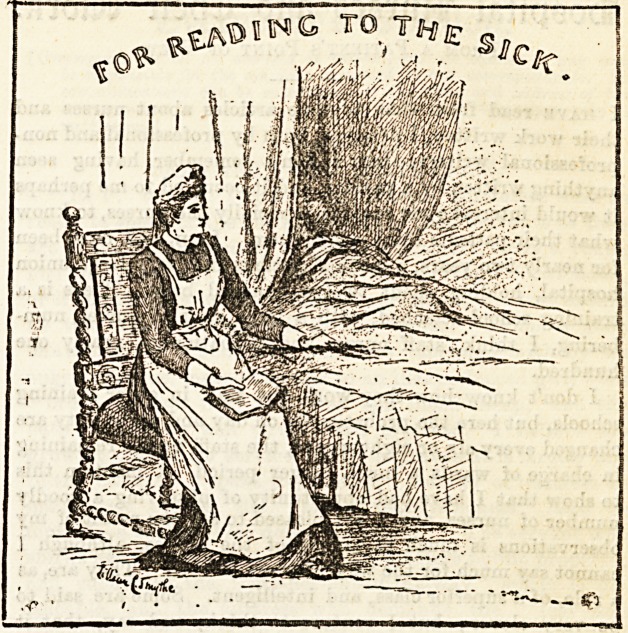# The Hospital Nursing Supplement

**Published:** 1891-06-13

**Authors:** 


### "The Hospital" Nursing Mirror.

Being the Extra Nursing Supplement of "The Hospital" Newspaper.

Contributions for this Supplement should be addressed to the Editor, The Hospital, 140, Strand, London, W.C., and should have the word "Nursing" plainly written in left-hand top corner of the envelope.

### En Passant.

ASYLUM ATTENDANTS.—Good news comes to us from Colney Hatch to the effect that 24 nurses have been up for the first examination of the St. John's Ambulance Association, and that 20 have passed. We give the names of the successful candidates: Nurses Ashfield, Alliston, Bovington, Barnes, Baker, Bryan, Cotterell, Dear, Ellis, Greenaway, Gosborne, Hillier, Kentsley, Kitchen, Lock, Mingay, Nelson, Smyrk, Welch, and Wise. The lectures were delivered by Dr. C. Theodore Ewart, the Assistant Medical Officer, and next winter he proposes to go on to the nursing course, and when that is completed, to adapt in eight lectures all the teaching to the special requirements of asylum life. Dr. Ewart firmly believes that skilled and trained attendants are worth double the number of untrained attendants, knowing that asylum physicians have to look for much of the success of their treatment to the reliability and character of the nurses. We hope to commence shortly a series of articles on asylum life, wherein the vexed question of the training of attendants will be fully dealt with. Meanwhile we congratulate the 20 successful nurses at Colney Hatch, and earnestly entreat them to go on learning from year to year, not only the technical part of their work, but that unfailing patience and quiet tact without which their labours are of little worth.

STEYNING AGAIN.—On April 4th we gave a short epitome of an enquiry which had been held with regard to the state of the Steyning Infirmary; the paragraph was headed "A Disgrace to England." But Steyning seems to revel in scandals, though we expect the last one is the result of a blunder and a misunderstanding, not of any wilfulness or neglect. However, at the last meeting of the Guardians the clerk read the following letter: "Dear Sir—I was requested by the foreman of the jury who held an inquest on the body of Sophia Jefferies, at the Steyning Union Workhouse, on Saturday, the 16th inst., to convey to the Guardians of the Steyning Union the following rider:—'That the jury are of opinion that the Head Infirmary Nurse, E. T. Von der Goltz, was negligent in the discharge of her duty in not having deceased, Sophia Jefferies, put to bed immediately after the bath given to her on the 15th instant.' The jury also thought better appliances for cleansing sick patients might be provided.—I am, Sir, your obedient servant, (Signed) Edward Bedford, Deputy Coroner." The Rev. A. D. Freeman said that when the woman was admitted to the House it was mentioned that she was suffering from ulcerated legs. Nothing was said about her being very ill at all. She was then taken to the Infirmary and treated in a very judicious, and proper, and considerate, and kind manner. Up to this time nothing at all was said about her serious illness. In mentioning the fact of there being no bed in the ward, which was the reason for the woman being kept in the chair so long, the rev. gentleman declared there was no bed vacant in spite of Mr. Gardner's remark that there was one, and, in conclusion, stated, in consequence of several facts, which were very material, not being brought before the jury, they were led to pass the rider. He asked them only to do justice to one who had worked hard and done good service. They were not going to undo the Coroner's verdict. The jury no doubt passed the rider upon the information before them, feeling justified in doing it, and they as Guardians now, under all the circumstances, ought to be prepared to do justice to one who had worked hard for them. The Chairman said he had no fault to find with the jury or the way in which the inquiry was conducted, but he had no hesitation in saying that, if he had had time to instruct the counsel he brought there, and he had been able to cross-examine the witnesses, that rider would never have been agreed to. On the other hand, the Medical Officer writes to the local paper as follows: "When the new nurse was appointed I was most anxious and willing to do all in my power to carry out the wishes of the Guardians; but I was prevented doing so by the action of the new nurse, who informed me that she had instructions to report all matters to the Committee and not to me, and when she took charge of the female side, together with Nurse Taylor, whom she herself engaged, she told me she was to take no instructions from me, but only from her; she was to do as she told her, and pay no attention to me, as I had sadly neglected my cases. How could it be expected under these circumstances that the work could be carried on without friction, however otherwise I might have wished it?" We expect the whole gist of the matter lies in this question of "friction" between the doctor and the nurse.

THE ROYAL RED CROSS.—The Queen has been pleased to confer upon Mrs. Grimwood the distinction of the Royal Red Cross, in recognition of her devotion to the wounded under most trying circumstances during the attack upon the Residency at Manipur. It is some years since this order was presented to any lady, and Mrs. Grimwood's services are scarcely similar to those which have formerly won the decoration. Probably if red tape had not interfered one of the Indian orders would have been conferred on Mrs. Grimwood, but unfortunately they are not given to women. Many of our readers must have seen Mrs. Grimwood's letter, which appeared in the daily papers, and described the fight and flight at Manipur, and the subsequent sufferings of the wounded which she did her best to relieve. One of the saddest sides of the subject was that the letter was full of affectionate references to her husband, who had been killed, though Mrs. Grimwood was, at the time, ignorant of the fact.

CUMBERLAND INFIRMARY.—The necessity for increased accommodation for the nurses of the Cumberland Infirmary has been before the public for a long time, and now an appeal has been circulated, signed by the Bishop. It says: "A strong opinion has been entertained by many friends of the Infirmary for some time past that it is necessary to make an effort to improve and increase the accommodation available for nurses. 1. It is the unanimous judgment of the medical staff that the accommodation for the nurses employed in the Infirmary is not up to the level now sanctioned by public opinion. The class of person available for hospital nursing has been altered, a larger number of nurses in proportion to the patients is required; and the fact appears to be that the Cumberland Infirmary is at this moment inferior to many others in the respect now under consideration. In order to give it that position which its supporters would wish it to occupy, and which, indeed, is necessary for its continued success and for ensuring the services of nurses of the highest class, it is necessary to erect sleeping accommodation, not in immediate connection with the wards, as in the present arrangement. 2. It is strongly felt by the visiting ladies, and representations have been made by them to that effect, that the present supply of night nurses is inadequate to the needs of the patients. It is thought that at least two more night nurses should be engaged; but the want of accommodation stands in the way of this improvement, and the provision of adequate lodging for night nurses is a second argument for increasing the existing buildings. 3. But further, it has been urged by the Matron, and her views have much commended themselves to many who have considered them, that it would be advantageous to the Infirmary, and a great benefit to the public at large, if the example set by many similar institutions were followed with regard to the supply of private nurses. 4. District nurses for the poor would be a great advantage.

### A Year in a German Hospital.

PART II.

In three weeks the boys' room was given me to manage under the Sister's supervision, besides two babies in cots. My duties were to carry out all the small dressings and bandaging prescribed, and to go round with the doctors. I very soon began to love the work, and to get very interested in the children. One very anxious case was that of a boy who had had the operation of trachæotomy performed for diphtheria, and, having thus had time to pull through and get quite well again, he came in to have the canula taken out. His name was Max, and he could only speak in a whisper, but was very clever at making us understand by signs what he wanted. First was inserted a smaller tube with a narrow opening and through which he could inhale but hardly exhale at all, as it had a flap, which closed at each breath. After a week or so, he was to keep a cork in the opening of the tube for a few minutes at a time, this gave him great labour in breathing, and to endure it at all he had to be kept well amused. If left alone for a minute he would pull the cork out, and laugh at us in his peculiar blowing fashion. It was quite to gain our own ends that we kept him in a good temper, as any excitement made it necessary to give him all the air possible. The first day the canula was taken away, and no tube inserted, he got frightened after a few hours, and was carried off blue and struggling to the operation room, the muscles being so strong as to nearly close the hole in one day. It was always a most trying time with diphtheria convalescents; and when I had night duty, I found how hard it was to insert even a small "*bougie*" tube into the trachæa when the child was convulsed with struggles to get breath, and was getting blue in the face.

One of my special cases was a baby, the eleventh child of its mother, and the only living one. It was a sickly, pathetic child, the very type of hospital babies in books; it never cried, was white and pretty, and died very soon. All its bones were diseased, and after excision of one shoulder and one hip-joint its temperature rose and reached 41 degs. centigrade, and the poor little thing died. One little girl of thirteen had been allowed to walk too soon after excision of the hip-joint, and had consequently grown on one side. She was straightened as much as possible under chloroform, and clothed in Gypsum from under the arms to the feet. She suffered torments from irritation of the skin, but was extremely good and patient, and was treated with great consideration by the other children, who brought her all their toys, and let her share in all the games she could.

I was in this ward two months, and got on very rapidly with technical expressions and German generally; it can't be hard to learn any language when you hear not a single word of your own, as was my case during my work there. Before going any further I ought to give a slight idea as to how the day was divided, so that English nurses may judge if we had long or short hours on duty. We had to be in the wards by six punctually, wash the patients, make beds, clear up, and give them coffee and a roll all round. At seven o'clock we went to our own coffee and roll, combined with prayers, said sitting, to save time. At ten minutes past seven we were back in the wards, swept and washed floors, and paint, and everything, making every preparation for the doctor's visit at ten o'clock. When they had finished in that ward, the patients had their breakfast, and when they were well provided for we had our meal, for which we were given twenty minutes. It consisted of bread and butter, with slices of cheese, or ham, or sausage, beer or milk, or seltzer water. I never could drink beer, though I took kindly to all the German food after a time. Returning after breakfast, we swept the ward again, and carried out any orders left by the doctors, and rolled bandages; and if the ward was a surgical one we prepared all that would be required for operations. At twelve o'clock we had half an hour to get ready for dinner. At this meal we invariably had soup, summer and winter. I surprised every one by not always caring for it, and by drinking a great deal of cold water, which they considered was very unhealthy. From one o'clock till half-past three we were in the wards giving out dinner, washing up, and going to operations. At half-past three, unless there was any special pressure of work, we had an hour to ourselves till we went to coffee at half-past four. After coffee we were in the wards till half-past seven, at which hour we had supper, that is, milk-soup, hot in winter and cold in summer, bread and butter, cold slices of meat, or ham, or sausage, and sometimes salad; beer again for those who liked it. After supper we could do as we liked. The Sisters all sat together, and worked or read; three of them used to spin. I generally sat in my own room, or with the other ladies. At nine o'clock prayers were read and sung in chapel, and then we retired for the night.

After my two months with the children, I was next promoted to the men's surgical ward, under a severe Sister, who taught one very thoroughly. It was summer time, and as the wards were being re-painted and done up, all the beds were moved into tents in the garden, which seemed to have a marvellous effect in healing wounds rapidly. I am bound to say the patients enjoyed it much more than the nurses, the latter had to carry all the hot water and food from the house, and very often got wet. The second afternoon I was to help at a big operation—cancer at the root of the tongue, extending sideways almost to the jaw-bone. The man was 52, and could not speak at all plainly. Tracheotomy was performed, and the jaw-bone sawed through, as the growth was very difficult to remove the operation lasted a long while. In a month the man went out, and very soon afterwards was back at his work. I was given the care of one tent with ten beds. One patient was recovering from hydatid, and though the disease was far advanced, and a whole pailful of bladders conaining white fluid (inside one huge bladder) were removed, he recovered splendidly. I saw three of these cases during my year, and helped at one of the operations, two out of the three recovered perfectly, the third, a woman, died of blood-poisoning through an accident. There were two cases of empyema, one of which was so far advanced when brought in that the patient died before the operation; the movement proved too much, and the disease reached the lungs.

Nearly all the disinfectants used were preparations of corrosive sublimate, the bandages, cotton wool, and moss-bags, all being steeped in a solution of it. It also proved a most effective cure for burns, and all flesh wounds to bathe the part in warm baths, of a weak solution twice daily for ten minutes at a time. Leg, arm, hip, and foot baths abounded in the surgical wards. Carbolic for dressings had been given up for years; it was only used in sprays, and for washing sponges during operations, or for floors and utensils in the wards.

We had some very interesting accidents and operations, which always seemed to come in batches, several on one day. On one afternoon, particularly, we had in three hours, one case of a broken leg, one of a pistol wound in the shoulder and neck, and another of a severe knife wound. All these were seen to in the operation room, and they had not all three recovered from the effects of chloroform when a youth of 21, was brought in deadly pale, and with a dreadfully torn arm. He was a butcher, and while hanging up some meat, the ladder he was on slipped, and he was left hanging by his arm on a meat-hook. He lived miles away from Berlin, and was taken first of all to a country hospital, where they told him his arm could not possibly be saved and must be amputated at once. He refused to have it done, and came straight to Berlin without even having it properly bandaged. He was almost unable to walk through pain and loss of blood, yet I have never seen anyone braver, or pain endured better. His arm was dressed and a long time it took without chloroform, all that night his temperature was up to 40 centigrade. The next morning the bandages were cut, and a great deal of flesh and some tendons were cut away, during which he showed no sign of pain except that he was quite white. The fever lessened, and in two weeks he was able to bathe the hand and arm; and though he never could recover the use of the thumb and the first two fingers, yet he lifted things with his arm, and went back to his business.

Another patient I got to like very much was a Pole, who had lived in London at "Wolff's Conditorei" in Ludgate Hill, as a confectioner; he had learnt a little bad English, and was thought very clever by the others when he exercised it for my benefit. He had already lost a finger in a biscuit machine, and this was the second, consequently he would find much difficulty in following his trade, especially as the second finger on his left hand had got stiff also.

I learnt massage very thoroughly from one of the doctors, and have since found it of great use in cases of indigestion, rheumatism, and contusions when the skin was not broken. We had two cases of badly bruised feet and sprained ankles; they had to keep the foot lying in a splint with ice bags on it for two or three days, after which it was massaged and bathed daily, of course very gently at first and for a short time; the patient might then use it as much as he liked. In seven or eight days no pain or discoloration was left. I always found the fellow patients most ready and willing to hold the limb for me, the first day or so of massage; they derived much amusement from the way one big strong man used to plead to have it rubbed very gently, and keep a sharp look out on the clock.

I certainly found the work hardest in this ward, owing, of course, to the necessary precautions against erysipelas, only one case of which occurred in the year. The ward was swept six times a day, and every day all the paint and furniture was washed. The men had plenty of games, and were allowed to smoke in the outside glass passage. They were extremely happy and willing to oblige, they were most modest and nice. The ward-man washed the ward daily, and attended to those wants and dressings which the Sister and ladies did not undertake, which I think made the men feel more comfortable, and kept a very good tone among them.

From this ward, which I was very sorry to leave, I went to the women's medical ward, and though the quiet routine work was not nearly so exciting, I think I learnt that which has been most useful to me in ordinary life since. When I had a case of typhoid to nurse I got quite absorbed. The patient was a young servant girl, and the fever kept steadily high; she passed the usual time for a change without being any better. Professor Senator was the head physician; he tried antipyrin in this case, with the effect of producing bad sinking symptoms; they were overcome by camphor injections every half-hour. Cold baths were also tried, with better success; but what the doctors said really told in such cases of long high fever was the punctual observance of the smaller treatments. Every two hours the patient was sponged over with salt and water and spirits of camphor, every half-hour small doses of medicine were given, and nourishment of some kind every quarter of an hour, the mouth being kept moist and clean with myrrh. Of course, there were special daily orders as well, but the former were done by the nurses to all typhoid cases. I was quite delighted when this girl recovered, and the few words the doctor said to me then gave me one of the greatest pleasures of my life. She was very grateful herself, and told me her history, which was very sad, though not an uncommon one.

### IMMORTALITY.

What a wonderful thing is life! The wisest among us can of himself tell nothing about its origin, but we, who believe in the Bible, know that it comes from God, that all things were made by Him and that "in Him was life."

"Heaven lies about us in our infancy," says the poet. "Trailing clouds of glory, we come from God who is our home." We feel the truth of all this when we think of the innocence of childhood, of the purity of the young when brought up like Timothy in the nurture and admonition of the Lord, and can see that as we near manhood we unhappily get farther from our Maker. Earth and the world give us so many pleasures of their own that at last we forget from whence we came. We soil our white robes with sin, and in sorrow, sickness, and despair cry "Lost, lost."

What comfort at such times to remember that we have that within which never dies, a germ that will live for ever and ever; that however far we have wandered from our home, into whatever depth of guilt or misery we have plunged, He who gave us the life can save us from the punishment of death. We have a soul that can never be annihilated, and though we read "the soul that sinneth it shall die," yet in the same place we find, "Christ came into the world to save sinners," and to bring back to the fold, to the home of happiness and light, the souls which have wandered away from His and our Father.

Jesus has invited us to drink of the living waters which He gives freely to all mankind without money and without price. Come to Him and drink freely, for He himself is that fountain which was opened for the healing of the nations, and which springeth up unto everlasting life.

Jesus lives! henceforth is death
But the gate of life immortal;
This shall calm our trembling breath,
When we pass its gloomy portal.

Jesus lives! to Him the throne
Over all the world is given;
May we go where He is gone,
Rest and reign with Him in heaven.

---

The After-Care Associations for Poor and Friendless Female Convalescents on leaving asylums for the insane, will hold its annual meeting at 83, Lancaster Gate, on June 15th. The Earl of Meath will take the chair at three p.m. Tickets can be obtained from H. Thornhill Roxby, Church House, Dean's Yard, Westminster.

### Hospital Nurses and Their Work.

From a Patient's Point of View.

I have read from time to time articles about nurses and their work written, I presume, both by professional and non-professional writers; but I don't remember having seen anything written by a patient, and it occurred to me perhaps it would interest your readers, especially the nurses, to know what their patients think about them. I am, and have been for nearly two years, a medical patient in a very large union hospital, holding about twelve hundred beds. There is a training school attached, with a large staff of nurses, numbering, I think, staff nurses and probationers, nearly one hundred.

I don't know how they work the staff in other training schools, but here the probationers on day and night duty are changed every six or eight weeks, the staff nurses remaining in charge of wards a much longer period. I mention this to show that I have had opportunity of observing a goodly number of nurses, and I am pleased to say the result of my observations is much in favour of the nurses, although I cannot say much for the way they are worked. They are, as a rule, of a superior class, and intelligent. Some are said to be very clever; but I venture to think, and say, that it takes something more than mere cleverness to make a nurse. I am of opinion that nurses will find they will require not only intellectual and professional qualifications, but if they want to be nurses of the true stamp they must really love their work and be in sympathy with their patients, and treat them with tender kindness and pity. My own experience is that I have always been much more benefited by medicine or food when it has been given me by a nurse who felt for me, rather than by one who gave it only because it was part of her work, and who did not care the price of a tinker's commission whether it killed or cured. The nursing profession is like other professions in that there is found occasionally in its ranks some very "queer fish." I don't think there are many Sairey Gamps and Betsy Priggs about now-a-days, but I have often had reason to wonder what in the name of goodness put it in the heads of some girls to take to nursing, because it is plain to everybody else, and ought to be to them, that they are altogether, physically and mentally, unfit for the work. Of this they ought to be convinced before the first month's training is finished. But they don't seem to see it; they keep on, apparently in happy ignorance that they are a source of trouble and anxiety to their superiors, and pain and misery to their patients. There is one type of nurse which is a complete puzzle to me. She walks into the ward in the same way we would expect to see a tailor's dummy do it, and goes about her work with as much intelligence as we would look for in that necessary but harmless image. She always takes care to let everybody know that she is there only for her own "pleasure," and she is altogether too utterly good for the work. In strong contrast to her is the blustering nurse who rushes into the ward like a locomotive into a railway station. She is generally a strong, hard-headed girl, and goes about her work with more strength than sense. When she is washing a patient's face you would think she was scrubbing a locker-top, and the way she combs his hair is a "caution." So long as she makes the patient *look* comfortable, it matters little to her how he *feels*. It is a melancholy sight to see a poor, half-dead patient trying to remove the superfluous soap and water from his eyes and ears after one of these "blusters" has made him "look" comfortable.

But the most objectionable nurse is, I think, the one that laughs and ridicules and sneers at her patients. Some do it openly and defiantly, others do it covertly and slyly, in an underhand way. They haven't a spark of feeling, and you look to them in vain for sympathy. The treatment of their patients is indifference carried to absolute contempt. Of course the patients don't set down quietly to that sort of thing, and consequently there is always bother and trouble wherever there is one of these objectionable creatures. These are only the "weeds" of the profession, and I have no doubt go the way of all weeds; but I can't help thinking whoever sends them to nurse patients incurs a very grave responsibility.

However, the majority of the nurses are quite the other way, being generous and warm-hearted, and going about their work intelligently and kindly. Many of them are quite in love with the work, and nurse their patients with much sympathy and tender kindness. Although I have heard that the work is only a means of getting bread and cheese for most of them, I haven't observed that they are neglectful because of that. The work is alike hard whether they work for love or bread, but I admit that I think that the nurse to whom it is a labour of love has the advantage. But whatever they may be working for, they have sometimes to work under trying circumstances, and what between narrow-minded officials on the one hand and ill-conditioned patients on the other, they often have a warm time of it. I think patients ought never to be surprised if nurse has a "pain" in her temper occasionally. There is hard work and long hours, and ingratitude and unthankfulness, for all who take to nursing, and I suppose they will have to put up with the conditions. But there is one thing all nurses can do, if they have a mind. Wheresoever their sphere of work may lie—nursing, or in the hospital, or district nursing—they are sure, at some time or other in their nursing life, to have patients who are wanting in force of religious character; or those who, through the threatening difficulties of the religious life, are becoming wearied and faint in their minds; or those in whom good and evil tendencies have long been contending for supremacy, and the good are well-nigh overmastered, and are ready to die. Or there may be some who have sinned away their best opportunities, and the sun is going down on the day of their visitation. Some may have procrastinated for so many years, that the time which remains to them is almost too brief for the great work of life. Among such the nurse, while she is doing her work of kindness and gentleness, can say a word of peace and hope for the Master's sake, and she may be sure every word spoken, every deed done in His name will be recorded above and placed to her account against the day of reckoning comes.

### Short Items.

At the request of the Marchioness of Waterford, a course of lectures on "Home Nursing" will be delivered by one of the National Health Society's lecturers at 30, Charles Street, St. James's Square, on Wednesday mornings.—Nurse Emily Hayes is leaving Boston Union Infirmary to be married. The guardians have elected an untrained nurse in her place.—At a general court at St. Thomas's, on Wednesday, the first piece of business was to receive a report of a letter from the assistant Secretary (railway department) of the Board of Trade, dated May 6th, 1891, stating that the Board had declined the application for a licence authorising the Royal British Nurses' Association to register as a limited company, without the use of the word "limited." Dr. B. Fenwick tried to raise a row about the Board of Trade refusal at the quarterly court of the London Hospital, but was beaten by 23 votes to nine.—We have received a warm letter in praise of the Brassey Home from Nurse Danter.—The presentation of the nurses' wedding present to Princess Louise Augusta, will take place at Buckingham Palace on June 26th.—The Second Thousand is made up, and they will probably be received at Marlborough House in the end of July.

### Examination Questions.

ONLY twenty-two answers were received to the last question, giving proof that the holidays are upon us, and that the questions had better stop till the autumn. The best answer was received from Nurse Headford, of Chelsea Infirmary, to whom we have sent Bell's "Notes on Surgery." Other answers worthy of notice were received from Nurse Fullwood, Sister Armstrong, Sister Dora of Dublin, A. E. Reddock, Sister Aldridge, A. Knaggs, and A. E. B. Williams. The following is the prize answer :—

SPECIAL DANGERS IN CONVALESCENCE FROM SCARLATINA AND TYPHOID FEVER.

*Scarlatina.*—The complications are numerous and important. Great care is required during convalescence, for even after a slight attack of the fever, serious diseases appear. Just as much care, therefore, is required after a mild, as after a serious attack. Because the disease has been had in a mild form, persons are apt to be too hazardous in resuming their ordinary mode of life, and serious results follow. The complications most likely to occur are :—(1.) Acute nephritis, characterised by albuminuria, hæmaturia, and dropsy. (2.) Rheumatic inflammation of the joints, which sometimes becomes so severe that suppuration ensues. (3.) Abscesses in the neck, and sometimes in other parts of the body, appear after scarlatina. (4.) Pericarditis. (5.) Inflammation of the internal ear, accompanied by a persistent discharge. (6.) Empyema.

SPECIAL DANGER FROM TYPHOID.

The convalescence from typhoid is gradual and slow and generally without serious drawbacks. (1.) Perforation. Until all danger is over in this respect, a patient should not be allowed to attempt to sit up or get out of bed. Death has been the result from so doing. After the ulcers have sloughed off, the bowels are so thin that the patient should be moved as gently as possible, or perforation is the consequence. (2.) Peritonitis. This arises through perforation. (3.) Bronchitis and pneumonia. (4.) Pleurisy. (5.) Constipation. An enema of oil is the safest to remove any accumulation in the bowels. Aperients should never be given except under orders from the doctor. Food should be carefully administered during the early stage of convalescence. Any rise in temperature should be noted, and fluid diet resumed for a time. Mental derangement or deafness may occur in some cases, but generally passes off after a time.

### Notes and Queries.

TO CORRESPONDENTS.—1. Questions or answers may be written on post-cards. 2. Advertisements in disguise are inadmissible. 3. In answering a query please quote the number. 4. A private answer can only be sent in urgent cases, and then a stamped addressed envelope must be enclosed. 5. Every communication must be accompanied by the writer's full name and address, not necessarily for publication. 6. Correspondents are requested to help their fellow nurses by answering such queries as they can.

### Answers.

*B. T.*—The Holland Institution, Monteé de Cinirey, Nice; it also has a branch at Mentone. Also Home for Invalid Ladies, San Remo, or Miss Pearson, Les Agaves, Cannes. There is no charge for asking queries.

*Vox Populi. E. de C., and Others.*—You break] our very simple rules for correspondents; we cannot insert letters written on both sides of the paper.

*M. H.*—We find that the institution you mention has never sent one of its monthly nurses to an infectious case, and as this casts doubt on your other statements, we cannot print your letter. We have gone fully into the matter.

*Constant Reader.*—There was an article, or articles, on "The Nursing of the Dying" in *Nursing Notes* two years ago. Doubtless the editor could find you the numbers if you inquire at the office, 12, Buckingham Street, Strand. A pamphlet on "Laying-out the Dead" was published by the Nursing Guild of St. Veronica, Worcester, about two years ago; price 3d.

### Everybody's Opinion.

*[Correspondence on all subjects is invited, but we cannot in any way be responsible for the opinions expressed by our correspondents. No communications can be entertained if the name and address of the correspondent is not given, or unless one side of the paper only be written on.]*

SERVANTS AND MISTRESSES.

"R. P." writes : I believe that if some mistresses were as ready to speak of the good qualities of their servants as they are of the bad, especially in giving characters, many a young girl would be kept from falling. If a servant is dismissed, or if she leaves a house by mutual agreement, the best should be told about her as well as the worst. Minus a good character, or a generally good one tampered with by an angry mistress, means idleness to an inexperienced girl, then "bread becomes dear and flesh and blood cheap," and the path of honesty and virtue hard to tread. I believe the growing dislike for service which is prevalent is largely due to the haughty "speak-if-you-dare" manner of mistress to maid. Of course, the "sweet reasonableness" *must* be mutual—a case of "set down the *we*, sir !" The servant who shirks her duties, makes household matters public property, and cultivates the "get-her-out-of-the-way" spirit is despicable; and the mistress who demands the "fear and trembling" service is, to say the least about it, unworthy of the services of good principled servants. I think the reasonable mistress who gets such poor returns for her "sweet reasonableness" is entitled to universal pity.

A HOLIDAY HOME.

"A NURSE" writes : Knowing the interest you take in the welfare of nurses, I venture to trouble you with a few lines. Some weeks ago I saw in THE HOSPITAL, an advertisement stating that nurses requiring change of air and rest could be received at Russell's Farm, Wethersfield, near Braintree, Essex, I have just returned from a fortnight's stay there, greatly strengthened, and refreshed in health and spirits, and charmed with the kindness and hospitality shown me by the Misses Parmenter, who manage Russell's Farm, and their father who owns it. Any nurse requiring relaxation, fresh air, country walks, and a warm welcome cannot do better than apply to the Misses Parmenter.

THE PLAIN PATH.

Though in the gloom we wander blind,
And duty's path be hard to find,
Hidden by brambles sharp and rough,
Ah ! surely tears are plain enough !
The sobs of sorrow and of pain,
The cries of them that cry in vain
For all our progress have no less
Of anguish and of bitterness.
Sorrow alone has endless reign,
Though faiths decay and empires wane ;
And we, who know not where to bow,
May find sufficient worship now,
If but one grief be turned aside,
Or one who thirsts be satisfied.
Come, let us go : at least we can
Serve the high God by serving man.

—*Sturm und Drang.*

### Wants and Workers.

[Under this heading, we propose to try for a few weeks whether we can be useful to our readers in making the wants of some known to others who are willing to do what work they can to aid the great cause of curing and cheering the sick.]

*Scraps Wanted.*—To make scrap books for children, and to cover a screen for lending. Old Christmas cards gratefully received.—*Nurse Winter, Stansted, Essex:*

*Poor Parish.*—A Queen's nurse could get letters for Rudloe Convalescent Home, Box, Wilts; particulars on application to the Matron.

*Boots.*—Nurse Barbara, Ellerslie, Tunbridge Wells, will send to any nurse for the use of a poor patient requiring them, a pair of size five women's boots, lace, wice soles, suit gouty or dropsical patient. Also pictures for scrap books, and some copies of THE HOSPITAL for 1890, to give away.

### Modern Superstitions.

The advanced, the intellectual, the intelligent woman is supposed to haunt the Somerville Club for Ladies in Oxford Street. Perhaps she does, but perhaps the existence of the advanced woman is only another *fin-de-siècle* illusion; anyway superstition is still rife in the ranks of the Somerville members. On Tuesday the ladies of the Club were lectured to on the subject of "Home Nursing" by Miss Mary M. Belcher. The lecture was not deep or learned, and considering the prevalence of influenza and the fact that nurses are not to be had in London just now for love or money, it was probably useful. Miss Belcher went so far as to speak about bed-sores and recommended ordinary precautions, such as rubbing in spirit, and ordinary treatment, such as providing water pillows, whereupon one of the audience arose and said that the proper treatment for bed-sores was to put a pan of water under the bed. Asked in what scientific way this prevented the patient's back from becoming abraded by pressure on the mattress, the member could not explain, but vouched for it that the treatment was always efficacious and was recommended by German nurses. This is not a bad specimen of the illogical feminine mind at all, but lest nurses should grow scornful we pass on to other examples in which we have known them to be the sinners. The monthly nurse is very fond of taking a baby away from the breast or the bottle every few seconds, sitting it upright and thumping its back; the process is supposed to "break up the wind," but we refuse to believe nature meant the wretched infants to be so treated. Again, District Nurses fall victims to stories about patent ointments, or the potato carried in the pocket to prevent rheumatism; some even fall so far as to bow down before Sequah. Therefore it behoves every nurse to ask the reason of any treatment told her by the laity, to be slow to believe, and to try to exercise that "rare" sense ridiculously called "common" sense.

### To Nurse Angela.

Weary from want of sleep,
 Wishing the day was done,
I lay in the hospital ward,
 Surrounded, yet strangely alone
When you came for an instant near me,
 And spoke in that low, sweet tone.

You looked so fair in your blue print gown,
 And the muslin cap on your head,
It rested my eyes to watch you;
 Sitting there close to my bed,
And all through that day and the night,
 I remembered the words you said.

It happened that not long after,
 When they thought I was nearly cured,
That the same old pain and sorrow
 Again had to be endured;
Then you came to be always with me,
 Till my fears were re-assured.

And through the long months that followed,
 As the weeks wore slowly away,
When life seemed to me so dreary,
 You were ever cheerful and gay;
Never a frown on your gentle face,
 As I watched you day after day.

And now the pain is all over,
 It is but a dream in my mind,
As I think of you, tenderly nursing,
 Those I have left behind;
And I wonder, if being a nurse,
 Would make me as patient and kind?

E. M. C.

### The Nurses' Bookshelf.

### NOTES ON SURGERY FOR NURSES.*

When a handbook reaches a third edition it is necessary to do little more than offer congratulations; the voice of the public has already given forth a final verdict, and rendered criticism superfluous. Bell's "Notes on Surgery" is a particular favourite amongst Edinburgh nurses, but deserves a wider recognition down South. It is the clearest of the many books on this subject, and it avoids the use of conventional language and technical terms; the sole aim being to help nurses to understand and perform their work in surgical wards.

### ANOTHER DICTIONARY.†

This is an abridgement of Mayne's Medical Vocabulary, and it does not strike us a particularly useful volume. Too much has been left out, and the definitions which are given are too technical. The price of the volume is its best point, but on the whole we would prefer to give 3s. 6d. for Keating's "Medical Lexicon." Perhaps when the present edition of this dictionary is sold out Messrs. Churchill may see their way to give a little more information in the same neat and handy form in which this volume is issued.

### A CONCISE PAMPHLET.‡

The promoters of the Midwives Registration Bill have issued what may be called a statement of their case in a very short and useful pamphlet. First there is a brief history of the movement, then a summary of the opinions of medical men and medical papers; a list of objections to the Bill, and the answers thereto. Considering the lengthy letters and discussions this movement has called up, it is refreshing to come across such a crisp and concise statement from which it is possible to glean facts without wading through masses of words.

* Notes on Surgery for Nurses, by Joseph Bell, M.D. Oliver and Boyd. Price 2s. 6d.

† A Short Dictionary of Medical Terms. Churchill. Price 2s. 6d.

‡ Notes on the Midwives Registration Bill. The Midwives Institute. Price 6d.

### Amusements and Relaxation.

**SPECIAL NOTICE TO CORRESPONDENTS.**

**Second Quarterly Word Competition commenced April 4th, ends June 27th, 1891.**

Competitors can enter for *all* quarterly competitions, but no competitor can take more than *one first prize* or *two* prizes of any kind during the year.

Proper names, abbreviations, foreign words, words of less than four letters, and repetitions are barred; plurals, and past and present participles of verbs, are allowed. Nuttall's *Standard* dictionary only to be used.

N.B.—Word dissections must be sent in WEEKLY not later than the first post on Thursday to the *Prize Editor*, 140, Strand, W.C., arranged alphabetically, with correct total affixed.

The words for dissection for this, the ELEVENTH week of the quarter, being

"OMNIBUS."

| Names. | June 4th. | Totals. | Names. | June 4th. | Totals. |
|---|---|---|---|---|---|
| Christie | 13 | 363 | Ivanhoe | 14 | 312 |
| Patience | — | 214 | Weta | — | 147 |
| Agamemnon | 15 | 367 | Lady Betty | — | — |
| Hope | 15 | 367 | Mortal | — | 76 |
| Reldas | )3 | 367 | Little Eliza | — | 147 |
| Lightowlers | 12 | 338 | Dove | — | 95 |
| Nurse J. S. | 15 | 310 | Ladybird | — | 141 |
| Qu'appelle | — | 170 | Psyche | 15 | 326 |
| Jenny Wren | 14 | 293 | Ugug | — | 229 |
| Wyameris | 15 | 369 | Harrie | — | — |
| Paignton | 12 | 306 | Grannie | 13 | 304 |
| Theta | — | 206 | Eale | — | 169 |
| Success | — | 17 | Grimalkin | — | 53 |
| Tired | — | 136 | Nurse G. P. | 13 | 91 |
| M. G. | — | 188 | | | |

**Notice to Correspondents.**

N.B.—All letters referring to this page which do not arrive at **140, Strand, London, W.C.**, by the *first post on Thursdays*, and are not addressed PRIZE EDITOR, will in future be disqualified and disregarded

## Figures and Tables

**Figure f1:**